# Portal Hypertensive Biliopathy Presents with Massive Bleeding during ERCP after Balloon Sphincteroplasty in a Noncirrhotic Saudi Sickler Patient

**DOI:** 10.1155/2017/4163919

**Published:** 2017-05-14

**Authors:** Ahmad M. Al-Akwaa, Mohammed Elsadig, Ahmed E. Al-Fayaa, Mohja D. Al-Shehri

**Affiliations:** Gastroenterology Division, Department of Medicine, King Abdulaziz Hospital, Al Hasa, Saudi Arabia

## Abstract

Portal hypertensive biliopathy (PHB) is described as abnormalities of the walls of the biliary tree secondary to portal hypertension. Gastrointestinal bleeding caused by PHB is rare. PHB as a cause of serious bleeding after sphincteroplasty during ERCP is extremely rare. Here, we report a case of PHB in a young Saudi male with cell sickle anemia who developed massive hemorrhage during ERCP after balloon dilation of the ampulla of Vater. We further discussed the diagnosis and management. To the best of our knowledge, no such case has been reported.

## 1. Introduction

Portal hypertensive biliopathy (PHB), also known as pseudosclerosing cholangitis, was first reported by Fraser and Broun in 1944 when they published a paper on an anomaly of the vena portal system [1]. However, the concept of portal biliopathy was fully described by Bhatia et al. in 1995 [2]. Treatment guidelines were formulated by Chaudhary et al. in 1998 [3]. Today, PHB is defined as a consequence of portal hypertension in which there is a development of both extra- and intrahepatic biliary ductal narrowing due to venous collaterals in or around the bile ducts, secondary to extrahepatic portal vein obstruction [4, 5]. While the majority of patients remain asymptomatic, 5–30% of cases develop symptomatic bile duct obstruction, cholelithiasis, and cholangitis [4]. Gastrointestinal bleeding caused by portal biliopathy is very rare. Portal hypertensive biliopathy as a cause of serious bleeding after sphincteroplasty during ERCP is extremely rare. To the best of our knowledge, no case has been reported. Here we report a case of PHB in a young Saudi male with cell sickle anemia who developed massive hemorrhage during ERCP after balloon dilation of the ampulla of Vater. We further discussed the diagnosis and management.

## 2. Case Report

A 23-year-old Saudi male, known case of sickle cell disease, presented to the medical OPD in June, 2013 with one-week history of mild vague abdominal pain in the left upper quadrant associated with early satiety. On examination, the patient was conscious. His vital signs were as follows: blood pressure 110/60 mmHg, pulse rate 84/minute, and temperature 37.1°C. He had mild pallor but not jaundiced. Abdominal examination revealed massive splenomegaly (10 cm below costal margin). His investigations were as follows: WBC: 4.32, Hb: 11.3, PLT: 184, LDH: 807, TBIL: 31, Conj.Bil: 0, ALT: 39, AST: 32, and ALKP: 66. Abdominal ultrasound showed homogenous echo pattern with slightly enlarged liver (16 cm at the right midclavicular line) and smooth hepatic boarders. There were no focal lesions; spleen was confirmed to be enlarged (20 cm) with dilated portal vein.

Ultrasound portal-splenic Doppler showed the diameter of the portal vein measuring 12.5 mm in caliber. It also showed normal patent biphasic flow pattern with average velocities and normal hepatopedal flow direction with no evidence of portal vein thrombosis. The hepatic veins also displayed average normal diameter measuring 10.5 mm in caliber. It appeared patent on color Doppler interrogation displaying average velocities with no evidence of portal-systemic collateral vessels. Hematology consultation recommended splenectomy which was carried out laparoscopically with uneventful postoperative recovery.

In July 2015, the patient presented to the OPD with obstructive jaundice. On examination, he was conscious, alerted, and oriented. His vital signs were as follows: blood pressure 112/58 mmHg, pulse rate 81/minute, and temperature 36.9°C. He looked jaundiced. Abdomen (after splenectomy) was soft, lax, and nontender. His investigations were as follows: WBC 5.9, Hb: 11.2, platelet: 251, BUN: 1.2, creatinine: 52, sodium: 138, potassium 4.2, chloride 105, bicarbonate 22. TBIL: 187.7, ALP: 425 AST: 84, ALT: 54, and Direct Bil: 42.5. Abdominal ultrasound showed gallstone with signs of cholecystitis, biliary dilatation, and common bile duct (CBD) stone. Abdominal Doppler ultrasound showed patent portal vein. The patient was admitted to the hospital where ERCP was performed; sphincterotomy and CBD stone extraction were done. He developed mild pancreatitis which resolved in two days.

**Figure 1:**
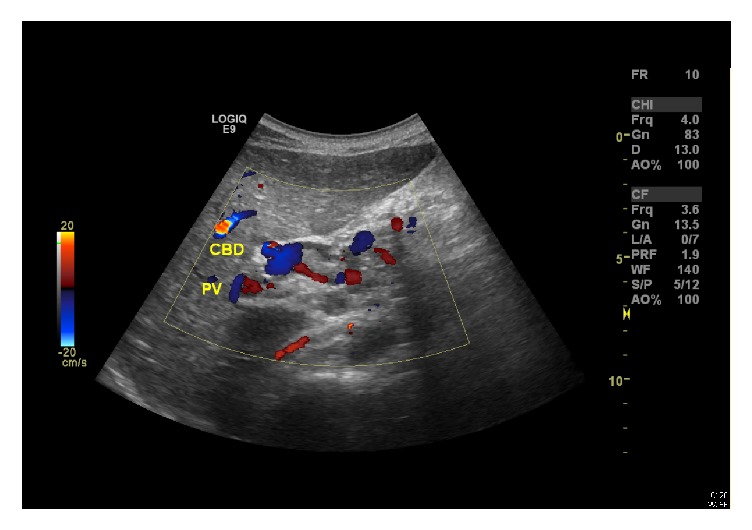
Abdominal ultrasound (post-ERCP with stenting) showing multiple intraluminal stones in the gallbladder with thick biliary sludge and diffusely thickened wall. Biliary stent is seen with the visualized portion of distal CBD that also appears slightly dilated at the porta hepatis. Minimal central intrahepatic biliary dilatation is also seen. Status after splenectomy.

**Figure 2:**
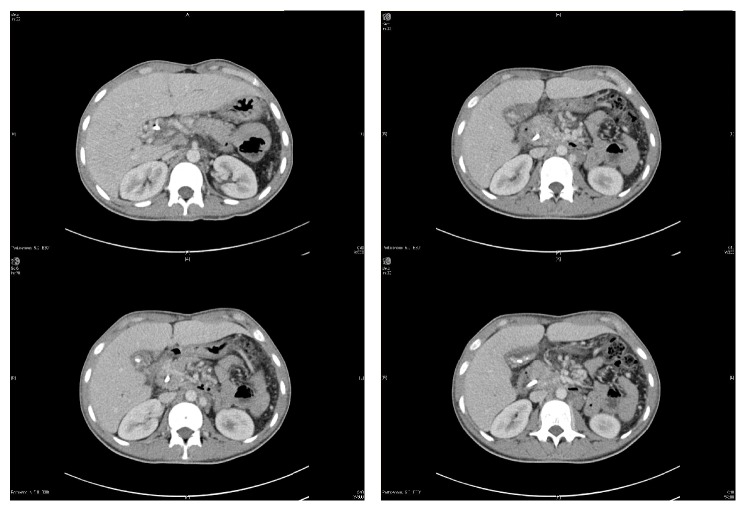
Multiple abdomen computed tomography (CT) scans showing the development of multiple collaterals in the porta hepatis replacing the portal vein, suggestive of cavernous transformation due to chronic portal vein thrombosis, in addition to multiple mesenteric and upper retroperitoneal collaterals. Multiple gallbladder stones and CBD stent are also seen. The liver is seen slightly enlarged with no detectable focal lesions. Normal enhancement of the hepatic veins. Normal size and parenchymal density of the pancreas. No detected gross masses. No detected calcification. Clear peripancreatic fat planes. Status after splenectomy.

In October 2015, the patient presented again with abdominal pain and obstructive jaundice. His full investigations can be seen in Table 1. Abdominal ultrasound showed gallstones, cholecystitis, and dilated bile ducts (Figure 1). He underwent ERCP; cholangiogram showed a CBD stone difficult to be extracted. Balloon sphincteroplasty up to 10 mm was performed which was complicated by massive bleeding from the ampulla. The blood was just oozing and pouring from the orifice. There was no arterial bleeding. The bleeding was massive and could not be controlled. The patient went into acute hypovolemic shock (Hb dropped to 5.8 g/dl), and he was then intubated and transferred to the ICU where he was resuscitated and received six units of packed RBCs and FFPs. His condition improved after two days. Later on, he was evaluated for the cause of massive bleeding. Abdominal CT showed cavernous transformation of portal vein with splenic and portal veins thrombosis, in addition to extensive collaterals at porta hepatis and around the biliary tree (Figure 2). Upper endoscopy showed grade II esophageal varices. ERCP was successfully performed after 2 days to assess the area of bleeding and put stent to assure free drainage of the bile duct. The patient's condition improved after ERCP. He was then transferred to the medical ward where his condition was stable with no bleeding or hemoglobin drop. His LFT results were as follows: TBIL: 58.1, ALP: 156, AST: 19, ALT: 13, TP: 72, INR: 1.3, and albumin: 33. The patient was seen by general surgery and planned for laparoscopic cholecystectomy.

## 3. Discussion

The majority of PHB cases are associated with extrahepatic portal venous obstruction, but only few patients are symptomatic [6]. According to a 2008 review study, the incidence of portosplenomesenteric venous thrombosis (PSMVT) after splenectomy is 3.3% [7]. In our patient, the imaging studies before splenectomy showed patent portal veins. Previous studies have shown more than 30 causes of PSMVT, with pancreatitis being the most common [8, 9]. The reported rate of pancreatitis-induced PSMVT is 16.6% [10]. However, it usually occurs in patients with necrotizing pancreatitis and is rare in the absence of necrosis [11]. Our patient had a mild attack of pancreatitis post-ERCP that had completely resolved in two days. We believe that the most likely cause of the massive hemorrhage after sphincteroplasty during the second ERCP is secondary to bile duct varices which was later seen on CT scan. Balloon dilatation is usually safe and rarely leads to bleeding. Studies reported the incidence of ERCP-associated bleeding to be 1.3% and the risk of severe hemorrhage (i.e., requiring >5 units of blood) was estimated to occur in fewer than 1 per 1000 sphincterotomies [12, 13]. In our case, the massive, uncontrollable bleeding occurred after a balloon dilation of 10 mm in the ampulla of Vater which was not the case of either arterial or small vessel bleeding. We believe that the bleeding was secondary to large tortious veins around the bile duct and ampulla of Vater.

The exact pathogenesis of portal hypertensive biliopathy is not completely understood. However, it is believed that external pressure of biliary venous plexus, portal cavernoma, and ischemic stricture of the common bile duct play a role [14].

PHB is initially investigated by abdominal ultrasound, which is helpful in detecting dilated/thrombosed portal veins, gallstones, cavernoma, and splenomegaly. Magnetic resonance cholangiopancreatography (MRCP) and endoscopic retrograde cholangiopancreatography (ERCP) are useful for assessing the length of the bile duct and the degree ductal narrowing; these noninvasive techniques aim at biliary decompression and reduction in portal pressure [15]. However, ERCP is the gold standard method for both diagnosis and treatment; it is usually used for therapeutic interventions [16].

Treatment is only indicated for symptomatic patients complaining of abdominal pain, jaundice, and fever. Biliary drainage and antibiotic therapy are the first line of management in cases of obstruction and cholangitis; some patients are treated surgically by portosystemic anastomosis. In persistent cases where patients remain symptomatic even after portal decompression, hepaticojejunal anastomosis with Roux-en-Y is considered [17].

Currently, there are no population-based studies on PHB. In Asia, there are a number of published reports with higher incidence in India and South Korea [18, 19]. In India, Layec et al. reported a similar case of massive hemobilia during extraction of a metal stent in a patient with PHB [20]. Our case differs in the fact that massive bleeding occurred after sphincteroplasty of the ampulla of Vater. The extension of the disease is lower in Europe and Americas. A recent study in Colombia reported 3 cases of noncirrhotic PHB [21]. None of them has a similar course as our patient.

Massive bleeding after balloon dilation of ampulla during ERCP can be secondary to PBH which has not been previously reported. This case demonstrates that we should be careful and prepared during ERCP in these kind of patients.

## Figures and Tables

**Table 1:** Laboratory data in October, 2015.

Variable	Normal value (SI) units	On admission
White blood cell	4–11 × 10^9^	6.55
Red blood cell	4.5–6.1 × 10^9^	4.84
Hemoglobin	13.5–18 g/dL	11.2
Platelets	150–400 × 10^9^	438
Total bilirubin	3.4–20.5 *μ*mol/L	204.6
Direct bilirubin	≤8.6 *μ*mol/L	151
Gamma-glutamyl transpeptidase	12–64 U/L	119
Serum alkaline phosphatase	40–150 U/L	461
Serum aspartate Aminotransferase	5–34 U/L	89
Serum alanine aminotransferase	5–55 U/L	59
Total protein	60–83 g/L	78
Serum amylase	25–125 U/L	109
Serum albumin	35–50 g/L	37
Blood urea nitrogen	3.2–7.4	1.1
Serum creatinine	64–110 mmol/L	53
Glomerular filtration rate	≥60 mL/min	181
Serum calcium	2.1–2.55 mmol/L	2.41
Serum phosphate	0.74–1.52 mmol/L	1.41
Serum sodium	136-135 mmol/L	135
Serum potassium	3.5–5.1 mmol/L	4.1
Serum lactate dehydrogenase	125–220 U/L	308
C-reactive protein	≤1.2 mg/L	18.3

## Abbreviations

PHB: Portal hypertensive biliopathy
ERCP: Endoscopic retrograde cholangiopancreatography.
